# A Review of Extraction Techniques and Food Applications of Flaxseed Mucilage

**DOI:** 10.3390/foods11121677

**Published:** 2022-06-07

**Authors:** Pradeep Puligundla, Seokwon Lim

**Affiliations:** Department of Food Science & Biotechnology, Gachon University, 1342 Seongnam-daero, Sujeong-gu, Seongnam-si 13120, Gyeonggi-do, Korea; puli@gachon.ac.kr

**Keywords:** flaxseed mucilage, extraction, food application, encapsulating agent, emulsifier, edible coating, fat replacer, stabilizing agent

## Abstract

Flaxseed contains significant concentration of mucilage or gum (a type of hydrocolloid). Flaxseed mucilage (FM) predominantly occurs in the outermost layer of the seed’s hull and is known to possess numerous health benefits such as delayed gastric emptying, reduced serum cholesterol, and improved glycemic control. FM is typically composed of an arabinoxylan (neutral in nature) and a pectic-like material (acidic in nature). Similar to gum arabic, FM exhibits good water-binding capacity and rheological properties (similar functionality); therefore, FM can be used as its replacement in foods. In this review, an overview of methods used for FM extraction and factors influencing the extraction yield were discussed initially. Thereafter, food applications of FM as gelling agent/gel-strengthening agent, structure-forming agent, stabilizing agent, fat replacer, anti-retrogradation agent, prebiotic, encapsulating agent, edible coatings and films/food packaging material, and emulsifier/emulsion stabilizer were included. At the end, some limitations to its wide application and potential solutions were added.

## 1. Introduction

Flaxseed, also known as linseed (*Linum usitatissimum*), consists of three layers, i.e., spermoderm, endosperm, and cotyledon from outermost to innermost. Flaxseed contains about 6% mucilage, and the spermoderm layer contains mucilage and lignans [1]. Flaxseed mucilage (FM), also known as flaxseed gum (FG), linseed gum (LG), or linseed mucilage (LM), is known to possess health benefits and has been used as a medicinally significant functional food (nutraceutical) for thousands of years. Soluble fiber from FM delays gastric emptying, improves glycemic control, and exerts a mild laxative effect or alleviates constipation [2]. The protective effect of FM on the mucous of the gastrointestinal tract has also been reported [3]. The ingestion of FG-incorporated wheat flour chapattis has been shown to significantly decrease fasting blood sugar levels and total cholesterol levels, especially low-density lipoprotein cholesterol, in type 2 diabetics [4]. Studies showed that, after receiving FG for 3 months, a significant reduction in the level of fasting blood sugar was noted in the experimental group (136 ± 7 mg/dL) compared to the control group (154 ± 6 mg/dL). In addition, the total cholesterol was decreased from 182 ± 11 to 163 ± 9 mg/dL within the experimental group.

## 2. Characteristics of Flaxseed Mucilage (FM)

Mucilages are hydrophilic in nature, and FM consists of two different types of polysaccharides, namely, a pectic-like material (acidic in nature) and an arabinoxylan (neutral in nature) [5]. The neutral fraction consists D-galactose, L-arabinose, and D-xylose in a molar ratio of 1:3.5:6.2. The existence of three distinct families of arabinoxylans (with a constant A/X ratio of 0.24, but varying in their galactose and fucose residues in the side-chains) in the neutral fraction of the FM has been shown [6]. Arabinoxylans of FM are composed of β-1,4-linked xylose backbones, which are either unsubstituted or substituted at the O-2 and/or O-3 positions by 1–3 sugar residues [7]. The acidic fraction consists of L-galactose, L-fucose, L-rhamnose, and D-galacturonic acid in a molar ratio of 1.4:1:2.6:1.7. The main chain contains all D-galacturonosyl units and all L-fucosyl, and about half of its galactosyl units are present as non-reducing end-groups [8]. The functionality of FM is similar to that of gum arabic [9]. Similar to guar gum, the mucilage exhibits good rheological properties and water-binding capacity (1600–3000 g of water/100 g of solids) [10]. FM has been shown to contain 50–80% carbohydrate, 4–20% protein, and 3–9% ash [10].

FG could form a thermo-reversible cold-set weak gel [11]. This weak gel-like behavior makes FG suitable to replace the majority of nongelling gums for nonfood and food applications [5].

An improved method (HPLC system using a column packed with Q Sepharose Big Beads™ gel and an evaporative light scattering detector) was developed by Elboutachfaiti et al. [12] for the fractionation (qualitatively) of flaxseed-derived water-soluble mucilage (WSM) polysaccharides into six acidic fractions (AWSM-1 to AWSM-6) and one neutral fraction (NWSM).

## 3. Extraction of Mucilage

**Figure 1:**
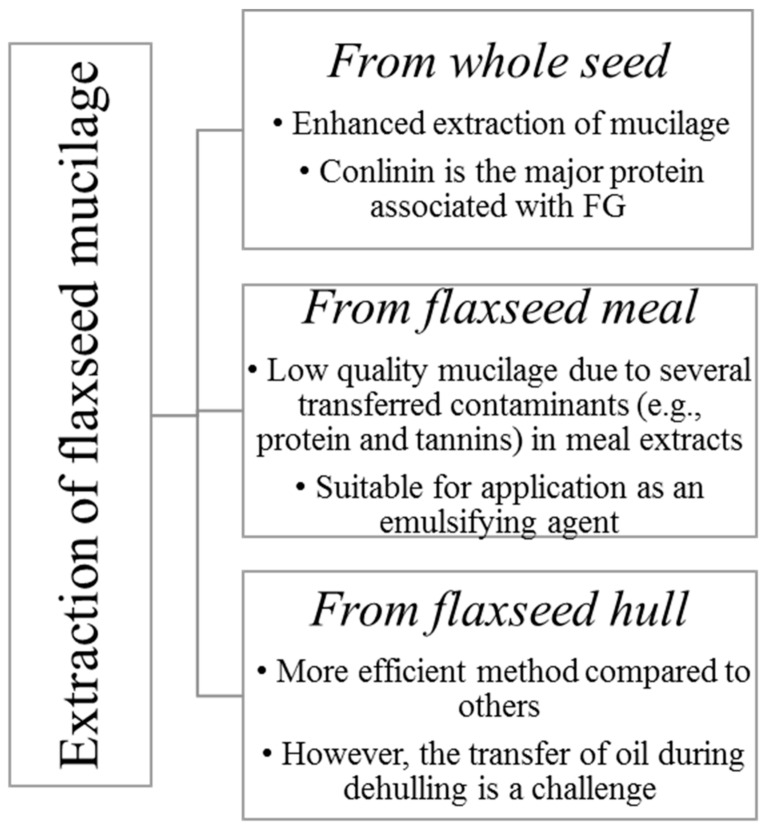
Different forms of flaxseed used for mucilage extraction (Adapted from Shang [14]).

Wet and dry processes can be used for flaxseed dehulling. Wet methods have been widely used for demucilaging of flaxseed [3,10,13]. FM is primarily obtained by aqueous extraction of the whole seed or meal or hulls [14] (Figure 1).

**Figure 2:**
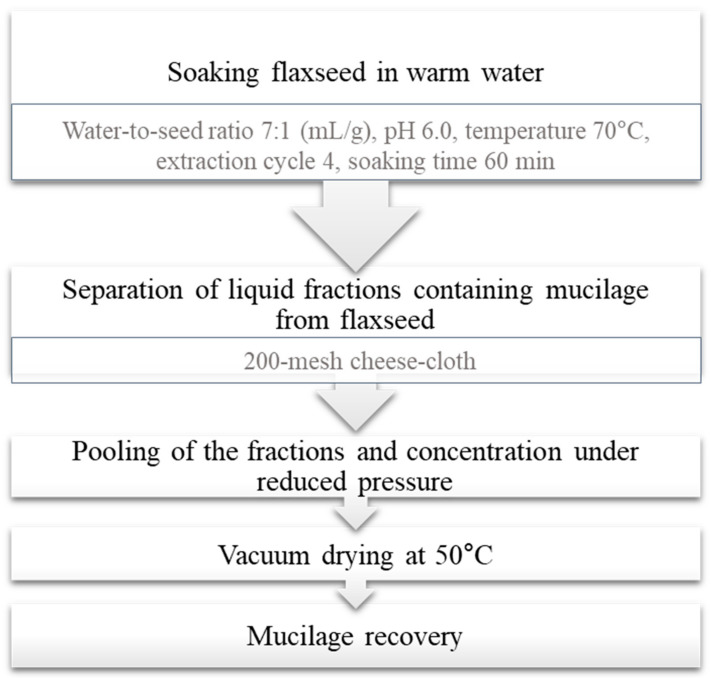
Typical steps in mucilage recovery from flaxseed via wet process (Reprinted with permission from Ref. Zhang et al. [1], Copyright 2022 Elsevier).

Different treatments of whole flaxseed, i.e., soaking in water or in 0.05 and 0.10 M sodium bicarbonate solution (for 6 and 12 h) or treatment with carbohydrases (Pectinex^™^ Ultra SP, Celluclast^®^ 1.5 L, and Viscozyme^®^ L), have been shown to remove a significant quantity of seed coat mucilage [15]. Zhang et al. [1] established a continuous wet process for dehulling and demucilaging of flaxseeds; typical steps in the recovery of mucilage fraction from flaxseed is given Figure 2.

Cui et al. [13] optimized the process of aqueous extraction of FG using response surface methodology (RSM). Their results showed that the optimal extraction conditions were a water-to-seed ratio of 13:1, a pH of 6.5–7.0, and a temperature of 85–90 °C. In another study, Ziolkovska [16] showed >99% extraction of water-soluble substances from the hull of whole flaxseed via the application of a three-stage countercurrent extraction process under the following conditions: a seed-to-water ratio of 1:25, a temperature of 80 ± 2 °C, and a duration of 30 ± 1 min (each stage). Rocha et al. [17] used a multi-objective optimization (MOO) method, namely, the normal boundary intersection (NBI), for FM extraction. They showed that, to obtain the highest fiber concentration in the extract, the optimal extraction conditions were a temperature of 46 °C, a pH of 3.81, and a time of 13.46 h; to obtain the maximum extraction yield, the optimal extraction conditions were a temperature of 65 °C, a pH of 6.45, and a time of 14.41 h.

Mucilages derived from different flax cultivars were shown to exhibit different rheological properties [18]. In solution, compared to its acidic counterparts, the neutral polysaccharide fraction (having a higher intrinsic viscosity) of FM exhibited more pronounced shear thinning and viscoelastic responses [10]. In the pH range 5.0–9.0, all the preparations of FM displayed stable viscosity; however, greater viscosity reductions were observed with the addition of electrolytes [10].

The extraction of FM is feasible commercially only as a byproduct of the flaxseed oil industry [12]. Extraction conditions (e.g., extraction temperature, pH) have a significant impact on the rheological properties, chemical composition, and yield of flaxseed gum [5,8].

### 3.1. Effect of Temperature

Extraction temperature can significantly affect FM structure, composition, and functional properties [19]. Compared with cold-water extraction, relatively higher yields of mucilage were obtained by the application of hot water as extractant; however, the preparation possessed high amounts of contaminating proteins [10]. The application of a clay adsorbent was suggested for the purification of mucilage, i.e., to remove water-soluble contaminating proteins. Upon increase of extraction temperature (from 30 to 90 °C), the quantity of denatured protein and monosaccharides in FG extracts increased, while the ratio of neutral to acidic monosaccharides decreased [19]. In addition, a significant reduction in the water absorption capacity and emulsifying activity index of FG has been observed with increased extraction temperature. In another study, hot-water extraction (HWE) was shown to be more advantageous than others (ultrasound-assisted extraction (UAE), alkaline–acidic extraction (AAE), and microwave-assisted extraction (MAE)) to improve the extraction yield of FG [20].

### 3.2. Effect of pH

In the FM extraction process, the pH of the extraction medium was shown to exhibit significant effects on extraction yield and soluble fiber content [21]. When FM extractions were carried out at different pH of 3.81, 6.75, and 9.69 for 13.25 h (at a temperature of 45 °C), the maximum yield was observed at pH 6.75. In addition, the highest content of soluble fiber was obtained at pH 3.81. Compared to other extractions, high-quality macromolecules were obtained via the extraction of flaxseed in basic medium (pH 9.69).

Hellebois et al. [22] used an alternative method to produce FG extracts from flaxseed husk at pH conditions close to the isoelectric point (pH ≈ 4.25) of flaxseed proteins. Gum extraction at pH ≈ pI did not enable the significant deproteinization of the gum extracts and did not diminish the residual protein content, ash content, and extraction yield. Moreover, the extraction did not alter the sugars and proximate composition of the gums. Adequate thermal and mild acidic pH stabilities were exhibited by all gum extracts, indicating their suitability for food applications. Compared to brown flaxseed gums, golden flaxseed gums displayed relatively higher molecular weight (M_w_ = 1.34–1.15 × 10^6^ Da) and intrinsic viscosities (6.63–5.13 dL·g^−1^), as well as superior viscoelastic and thickening performance.

### 3.3. Ultrasonication

Ultrasonication was shown to be a highly efficient method for the aqueous extraction of FM compared to other methods, namely, using microwaves and magnetic stirring [23]. Using ultrasonic treatment, an extraction time of 30 min was adequate to realize the quantitative extraction of the mucilage. The viscosity of extracted mucilage decreased with increase in ultrasound amplitude. The ultrasonic treatment exerted a limited impact on monosaccharide composition and protein content. Safdar et al. [20] showed that UAE was favorable to the purity of FG. UAE-FG showed a significantly higher scavenging ability in terms of 2,2-azino-bis-3-ethylbenzothiazoline-6-sulfonic acid (ABTS) free radicals, 2,2′-diphenyl-1-picrylhydrazyl (DPPH) free radicals, reducing power, and the β-carotene bleaching assay than HWE-FG, MAE-FG, and AAE-FG. For the optimization of ultrasound-assisted extraction of FG, response surface methodology (RSM) was applied by Akhtar et al. [24]. They showed that sonication extraction factors, namely, water-to-meal ratio, sonication extraction temperature (°C), sonication extraction pH, amplitude level (%), and sonication extraction time (min), had a significant impact on the gum yield, which varied in the range of 7.24% to 11.04%.

### 3.4. Extrusion and Enzymes

Extrusion and enzyme (e.g., Pectinex Smash XXL) treatments have been used for the removal of FM [25]. Rheological characteristics of FM under varying extrusion conditions with or without enzyme treatment were studied by Wu et al. [26]. Their results showed that extrusion could significantly degrade the FM, and the apparent viscosity of the mucilage decreased with increasing enzyme loading amount and hydrolyzing time.

### 3.5. Purification

Warrand et al. [27] purified water-soluble three distinct polysaccharides from FM using a combination of ion-exchange and size-exclusion chromatography. Among these, the major polysaccharide (75%) was a neutral polymer with a Mw of ~1.2 × 10^6^ g·mol^−1^. The two others were acidic polysaccharides designated as AF_1_ (3.75%, 6.5 × 10^5^ g·mol^−1^) and AF_2_ (21.25%, 1.7 × 10^4^ g·mol^−1^). A modified coprecipitate method was recommended for developing a low-protein high-mucilage product (M2) from detoxified flaxseed meal (optimum conditions for its preparation were a meal-to-water ratio of 1:40, temperature of 5 °C, and mucilage-to-ethanol ratio of 1:50) [28]. M2 exhibited good foam stability and emulsifying properties; therefore, it can readily be used for fortifying bakery products, pastas, etc.

## 4. Food Applications of FM/FG

**Figure 3:**
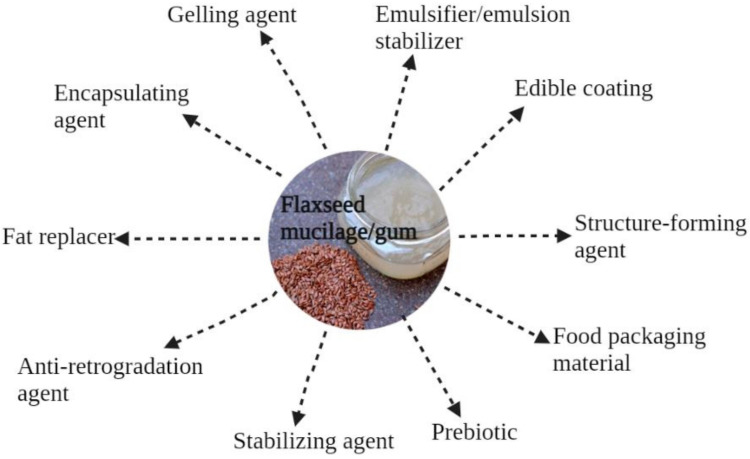
Potential food applications of flaxseed mucilage.

The utilization of mucilage gums depends on their distinctive functional properties, including gelation and water-binding properties, emulsifying and foaming properties, and viscosity, as well as on their bioactive role in the treatment and/or prevention of certain diseases [29]. FM can be used as a gelling agent [11] or thickener [26], and it possesses restricted foaming properties [30,31,32]. Typical food applications of FM are shown in Figure 3.

### 4.1. As Gelling Agent/Gel-Strengthening Agent

Due to its weak gelling properties, soluble FG is limitedly used as a gelling agent in food [11,33]. In a study, the gelling capacity of FG was improved by mixing it with agar (AG) and konjac glucomannan (KGM). Hydrocolloid gels consisting of FG, KGM, and AG in a ratio of 8:2:0.5 exhibited relatively high structural strength, high elasticity, and better water retention capacity [34]. Yang et al. [35] developed a novel gelling agent composed of FG, KGM, and AG; compound gels prepared using the mixture (FG, KGM, and AG at a ratio of 4:6:2) exhibited relatively better elasticity, good water retention capacity, and rigid surface morphology.

Phenolic antioxidants (in larger doses) can be used as alternatives to synthetic antioxidants to impede oxidation of meat proteins. However, it was shown that such high doses of phenolics deteriorated the gel-forming ability of myofibrillar proteins (MP) [36]. FG was used to decrease the negative effect of phenolics (catechin) on the gel characteristics of MP, which in turn enhanced the quality of meat products [36]. The inclusion of FG improved the dynamic rheological properties, water-holding capacity, and gel strength; the improved gel properties could be attributed to the increased hydrophobic interactions and disulfide crosslinking during gelation, as well as the formation of stabilized and uniform emulsions with high apparent viscosity. Chen et al. [37] showed that FG can be used as a thickener in a food system containing peanut protein; an increase in the apparent viscosity of FG–peanut protein isolate (PPI) dispersions with increasing FG concentrations (1–5 g/kg) was observed. The presence of FG can increase the gel strength and decrease the gelling time of heat-induced PPI gels.

### 4.2. As Structure-Forming Agent

Hydrocolloids such as methylcellulose (MC), hydroxypropylmethylcellulose (HPMC), carboxymethylcellulose (CMC), oat β-glucan, guar gum, pectin, xanthan, carrageenan, konjac gum, agar, locust bean gum, and psyllium gum are commonly applied as gluten replacers (or as structure-forming agents) in gluten-free products [38,39]. Linseed mucilage (especially freeze-dried mucilage) was successfully used as a natural structure-forming agent, replacing guar gum and pectin, in gluten-free bread [39]. The rheological properties of the dough with mucilage were comparable to those of a starch–pectin–guar gum system. Compared to control, the use of the linseed mucilage exerted a minor impact on texture characteristics and crumb staling of the bread. In addition, the addition of linseed mucilage (especially at 1.8% and 2.4% concentrations) improved sensory acceptance of the bread.

### 4.3. As Stabilizing Agent

Since FM closely resembles gum arabic, it can be used as a replacer of gum arabic in emulsions. Mucilage displays high solubility and satisfactory foam stabilizing properties in aqueous solutions. A 1% (*w*/*v*) linseed mucilage solution displayed foam values ~75% of those of ovalbumin at the same concentration [3]. However, at a slightly higher concentration of the polysaccharide (1.5%), the foam displayed a reduction in volume due to the compactness of the foam [40]. This behavior was due to the fact that the inclusion of small quantities of FM imparted some viscosity to the aqueous dispersion, and exerted a beneficial effect on surface-active proteins, whereas, at higher concentrations, the viscosity increased significantly, which reduced the free expansion of the foam [3].

The addition of FM at a level of 0.025% was shown to enhance the sensorial and physical characteristics of ice cream [41]. The potential of FM as a natural stabilizer for enhancing the texture of stirred yogurt was shown by Basiri et al. [42]. In their study, the inclusion of FM and FM + CMC in stirred yogurt decreased the syneresis and increased the viscosity. The addition of FM (0.6 g/100 mL) increased the adhesiveness and decreased the cohesiveness of the stirred yogurt, while its combination with CMC (0.6 g/100 mL FM + 0.3 g/100 mL CMC) led to increased springiness and cohesiveness, as well as decreased adhesiveness. However, sensory characteristics were not significantly affected by FM and FM + CMC supplementation during storage at 4 °C for 21 days. Akhtar et al. [24] showed that the incorporation of flaxseed-derived polysaccharide gums as stabilizer into yoghurts (at 1% level) improved their sensory and rheological properties.

The addition of FM as a non-starch polysaccharide has been shown to improve physicochemical and sensory characteristics of wheat flour noodles [43]. They showed that the inclusion of FM at a concentration of 3% and drying within the temperature range of 68.2–70 °C were the best conditions for preparing the noodle with high cooking quality compared to the control (100% wheat flour-based noodle). Additionally, compared to the control, the substitution of wheat flour by FM enhanced the textural quality and overall acceptability of prepared noodle.

### 4.4. As Fat Replacer

FM as a prebiotic and fat replacer was used to develop a fat-free and probiotic-fortified cream cheese [44]. The inclusion of FM in the cream cheese decreased the moisture content and the pH value, while the concentrations of total solids, protein, and ash were increased. A significant increase in the viscosity of cream cheese was noted with the addition of FM; however, a reduction in the viscosity was noted during the storage period. The application of FM in combination with probiotic bacteria exerted bactericidal activity against a few pathogenic bacteria. The FM inclusion enhanced the survival of the probiotic bacteria, improved the texture, and increased the overall sensory quality of the cheese. Another study showed the potential of FG and whey protein microparticles (WPMs) as fat substitutes in model mayonnaises [45]. Both FG and WPMs improved the textural properties (e.g., adhesiveness, cohesiveness, hardness, and consistency), rheological properties (e.g., storage modulus and viscosity), and kinetic stability of model mayonnaises; this effect was largely ascribed to the inclusion of FG, which likely formed an entangled 3D network. The texture of model mayonnaises was significantly influenced by FG; sensory scores for mouth-coating and creaminess increased with increasing FG concentration, whereas those of spreadability, fluidity, and firmness decreased. In the development of low-fat mayonnaises, the synergistic role of FG and WPMs was highlighted in the study. Hijazi et al. [46] used the FG obtained from cold-pressed flaxseed oil byproduct in a reduced-fat vegan mayonnaise formulation as emulsifier and fat replacer. Their results showed that FG may provide desired microstructural and rheological properties, as well as oxidative stability in the development of low-fat vegan mayonnaise. El-Sayed et al. [47] used FG in the range 25–100% to replace fat in cakes. A 100% fat replacement using FG resulted in 21.11% reduction in calorie content of the cakes. Additionally, FG addition (up to 75%) imparted resilience and tenderness to the cakes; fat replacement at this level resulted in cakes with highly acceptable sensorial properties.

Other plant seed mucilages such as chia mucilage (CM) have been shown to be suitable for fat replacement in mayonnaise, ice cream, and yoghurts [48]. Different studies have shown that CM can potentially be used as a fat replacer at up to 30% fat substitution in cookies and 25–50% in cakes and bread without negatively affecting the sensory qualities of final products [48,49].

### 4.5. Anti-Retrogradation Agent

Retrogradation negatively affects the consumer acceptability and quality of starchy foods. To control starch retrogradation, hydrocolloids such as corn fiber gum and guar gum have been used. Yang et al. [50] showed the retardation of retrogradation of maize starch (MS) upon the addition of FG. However, when FG concentration reached 0.4%, it caused no statistically significant increase. Feng et al. [51] showed that MS retrogradation was inhibited by FG addition under varied storage temperatures. Upon the addition of FG to the starch gels, the formation of hydrogen bonds with starch molecules could have occurred and absorbed more water to inhibit the rearrangement and recrystallization of starch molecules (amylose and amylopectin), thereby decreasing the water loss of starch gels and enhancing the texture of the gels. The impeding effect of FG on the retrogradation of MS is linked to good water-holding capacity and gel properties of FG.

### 4.6. As Prebiotic

Lai et al. [52] showed the prebiotic potential of FM. Compared with other tested mucilages, FM exhibited relatively more resistance against hydrolysis by acid and pancreatin, with a relative prebiotic score of 98%. FM promoted the growth of *Lactobacillus rhamnosus* GG. Therefore, the potential prebiotic capability of FM and its symbiotic relationship with *L. rhamnosus* GG suggest that they can be incorporated simultaneously for the production of functional foods.

### 4.7. As Encapsulating Agent

The protective ability of FM toward *L. rhamnosus* GG during microencapsulation and transition through the gastrointestinal tract was shown by Lai et al. [53]. Microbeads containing the *L. rhamnosus* with FM in the wall and core materials exhibited the highest encapsulation efficiency (98.8% *w*/*w*), a smooth surface (with 781.3 µm diameter), lowest erosion ratio (515.5% *w*/*w*) and swelling (5196.7% *w*/*w*), and lowest cell release (<40% *w*/*w*) with 9.31 log_10_ CFU·mL^−1^ after sequential digestion.

The storage stability of microencapsulated (using FM as a component) *L. rhamnosus* GG in hawthorn berry tea was determined by Lai et al. [54]. The microencapsulates were prepared with alginate–pectin–flaxseed mucilage as wall material and FM as core material. Under simulated gastrointestinal digestion, the encapsulated bacteria exhibited the highest viability (7.5 log_10_ CFU/mL) after 4 weeks of storage at 4 °C. They concluded that the optimized *L. rhamnosus* GG microbeads with FM could improve the functional quality of hawthorn berry tea.

FM was used to co-encapsulate probiotic *Lactobacillus casei* on an alginate (ALG) matrix [55]. *L. casei* growth in MRS broth was positively affected by FM, and the maximum viable count of the bacteria was obtained by using 0.9% FM. The inclusion of FM during microencapsulation enhanced the tolerance of the *L. casei* against the adverse effects of the simulated digestive system. During passage through simulated gastrointestinal conditions, the lowest reductions in viability were seen with 2% ALG + 0.9% FM and 3% ALG + 0.9% FM.

A tertiary conjugate was fabricated using FM, gelatin, and oxidized tannic acid [56,57], and it was found that the complex (FM–gelatin–oxidized tannic acid) could be used for the protection of oxidation-sensitive food ingredients and controlled release of bioactives in delivery systems in the food industry. A tertiary conjugate of gelatin–FM–oxidized tannic acid (OTA) was used (as wall material) to encapsulate flaxseed oil (which has a high amount of unsaturation) [56]. It is well known that, compared with saturated oils, oils with a high degree of unsaturation are highly susceptible to oxidation. The complex successfully protected and improved oxidative stability index of flaxseed oil, while exhibiting controlled-release ability. During storage, encapsulated oil exhibited a relatively low peroxide value compared with free flaxseed oil. Hadad and Goli [58] demonstrated that, for encapsulation of flaxseed oil, FM nanofiber can be used as wall material. The highest loading capacity was observed in nanofiber containing 40% (*w*/*w*) flaxseed oil. Lipid oxidation parameters indicated that the stability of entrapped flaxseed oil in the nanofiber was enhanced compared to the unprotected oil.

For spray drying encapsulation of *Lactobacillus acidophilus* La-05, FM and flaxseed soluble protein were applied as wall materials [59]. The flaxseed-based encapsulating wall protected the probiotic bacteria during spray-drying, as well as under simulated gastric conditions. Spay-drying conditions including 0.2% *w/v* of FM and an inlet air temperature of 110 °C during drying were shown to be optimal for maximizing (78%) the survival of the bacteria.

In the food industry, complex coacervation (a microencapsulation technique) can be used to protect volatile and unstable food ingredients (e.g., flavors, enzymes, vitamins, and ω-3 and ω-6 fatty acids) against oxidation and/or degradation [60]. It is a phase separation process resulting from electrostatic interactions between anionic polysaccharide and protein in an aqueous medium when the pH is below the isoelectric point (IP) of protein. Different studies showed the potential of FG as a polysaccharide of complex coacervates used for the encapsulation of unstable/volatile compounds (Table 1).

To encapsulate flaxseed oil (a rich source of ω-3 fatty acids), flaxseed protein isolate (FPI) and FG coacervates were successfully used as wall material [61]. Pham et al. [62] showed that complex coacervates developed using FG and polyphenol-adducted FPI were superior to FPI/FG complex coacervates as wall materials for encapsulation of oxygen-sensitive oils (Table 1). FG and rice bran protein (RBP) were successfully used to form complex coacervates [60]. The formed coacervates exhibited thermal stability and, therefore, can be appropriate for encapsulating heat-sensitive compounds. Hasanvand and Rafe [63] used RBP–FG coacervates as wall material for the encapsulation of vanillin/β-cyclodextrin inclusion; controlled/sustained release and high thermal stability of vanillin were achieved through the microencapsulation, and these microcapsules can be used in pastry and dairy products.

### 4.8. As Food Packaging Material/Edible Coatings and Films

Because of its film-forming ability and biodegradability, FG is used for preparing edible coatings. Potential for the development of edible films using FM was shown by Tee et al. [64,65]. Glycerol was added as a plasticizer to overcome the brittleness of the films. They showed that chemical interactions (probably hydrogen bonding) between the glycerol and the FM contributed to the improved properties of the developed films. In addition, glycerol-plasticized FM films exhibited an *a*_w_ value of 0.59 or less, which is unfavorable for microbial growth. The developed films exhibited sufficiently high oxygen permeability (OP) and water vapor permeability (WVP); therefore, these can potentially be extended for packaging or coating fresh produce. Tee et al. [64] suggested that glycerol can be added (up to 5 wt.%) as a plasticizer into the FM-based edible film. The film formation failed at 6 wt.% glycerol inclusion. 

In general, films made from pure mucilage lack the adequate mechanical properties to fit the food packaging needs. Other limitations to the use of FM in food packaging include high viscosity, high water solubility, and high degree of swelling. One of the effective ways to enhance the physicochemical properties of FM coating is blending modification [66]. It has been shown that, compared with films made from pure mucilage, films produced from the blend of FM and polyvinyl alcohol (PVA) (at a ratio of 1:1) exhibited less resistance, less rigidity, more flexibility, high thermal stability, and unaltered water vapor barrier properties [67]. Prado et al. [68] demonstrated that the degree of water solubility and the swelling of FG-based films were reduced due to the incorporation of cellulose nanocrystals, while the stiffness and tensile strength of the films were remarkably improved. Recently, Yang et al. [66] prepared three-layer FG/chitosan (CS)/FG coatings containing laurel essential oil and eugenol via a casting method, and they were used to preserve the myofibrillar protein in rainbow trout fillets. These composite coatings exhibited better mechanical properties than FG coating, and they possessed relatively high DPPH radical removal capacity, as well as total antioxidant capacity. The oxidation of protein in the fish fillets was inhibited, and the protein secondary structure was stabilized by the composite coatings. It was concluded that the composite coatings can potentially be used in food preservation.

Chitosan–FM nanofibers enriched with the essential oil of *Ziziphora clinopodioides* (ZEO) and sesame oil (SO) were prepared using an electrospinning approach [69]. These fibers exhibited good antimicrobial and antioxidant properties; they can potentially be used in food packaging applications for ZEO and SO encapsulation and for their sustained release.

In another study, Karami et al. [70] examined the structural, physicomechanical, and morphological characteristics of chitosan (CH)–FM films enriched with SO (0% and 0.75%) and ZEO (0%, 0.25%, and 0.5%), and they studied the effects of these films on minced trout fillet preservation. They showed that vacuum packaging (VP) and modified atmosphere packaging (MAP; 40% N_2_ + 60% CO_2_) enhanced the shelf life of the trout samples packaged in CH–FM films with SO (0.75%) and ZEO (0.25% and 0.5%). Following 16 days of refrigerated storage, treated samples were found to contain relatively low microbial counts (0.35–4.91 log CFU/g lesser) compared to the controls. All chosen treatments exerted no adverse effects on sensory characteristics of the samples. The levels of peroxide value, total volatile base nitrogen, and thiobarbituric acid reactive substances (TBARS) of the treated samples with SO 0.75% + ZEO 0.5% films were relatively low compared with untreated samples.

Edible coatings (ECs) based on FM for food preservation are gaining increased attention. Some reports of successful application of flaxseed mucilage as edible coating on food materials are given in Table 2.

**Figure 4:**
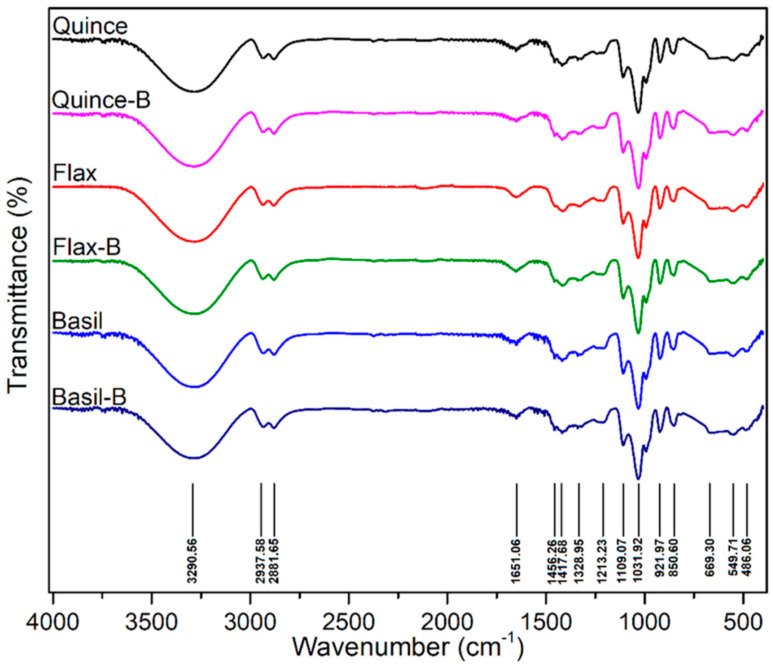
The FTIR spectra of films without LGG and with LGG after 1 month in 4 °C. No noticeable difference was observed between the samples (Figure reproduced from Davachi et al. [78]).

Davachi et al. [78] fabricated edible films based on flaxseed and other (quince and basil) seed mucilages with lactic acid probiotic strain *L. rhamnosus* GG (LGG). All of the mucilage films exhibited a high moisture retention capacity irrespective of the presence of probiotics, which is desirable for preserving the moisture/freshness of food. Compared with basil mucilage films, flaxseed mucilage films exhibited relatively high thermal stability and mechanical robustness with higher elastic moduli and elongation at break. These films effectively maintained the viability of the probiotics and protected fruits against UV light during storage. All the films, which were obtained from different seed mucilages, exhibited a similar polysaccharide structure, and the presence of bacteria (over a month) did not affect the structure of the samples, as shown in Figure 4.

**Figure 5:**
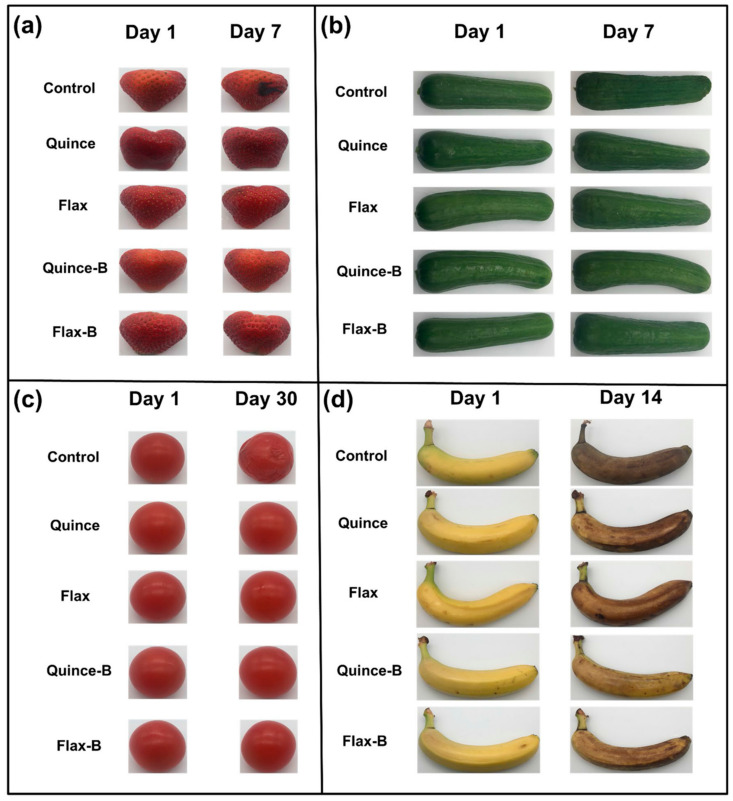
(**a**) Strawberries, (**b**) cucumbers, (**c**) cherry tomatoes, and (**d**) banana coated with quince and flax with and without probiotic LGG as compared with the uncoated samples (Figure reproduced from Davachi et al. [78]).

In addition, coated fruits and vegetables retained their freshness longer than uncoated produce, as shown in Figure 5.

Tabibloghmany et al. [79] showed that linseed hydrocolloid can be used as an edible coating for reducing oil absorption in potato chips during deep-oil frying. All tested concentrations (1.5%, 2%, and 2.5%) of aqueous solutions of the hydrocolloid were found to be efficacious in reducing oil absorption; however, the maximum effect was noted upon application of the solution containing 1.5% linseed hydrocolloid. Treatment with the solution (1.5%) was the best in increasing moisture content of potato chips, in reducing oil uptake, peroxide value, and acidity of extracted oil, with no significant alterations in texture when compared to the control samples.

Fang et al. [77] developed edible films based on FG and sodium alginate (SA) with varied concentrations of carvacrol and used these films as coating on the surface of Chinese sea bass fillets during cold storage. Fang et al. [80] applied sonication in the preparation of FG–carvacrol films. Results showed that sonication at different powers improved the distribution of carvacrol (i.e., a more homogeneous dispersion) in the FG matrix. The tensile strength (TS) and elongation at break (EB) of the FG–carvacrol films were increased by sonication. In addition, sonication led to the enhancement of antibacterial properties of the films against *Pseudomonas fluorescens, Vibrio parahaemolyticus, Staphylococcus aureus,* and *Shewanella putrefaciens,* as well as improved antioxidant properties.

FG, in combination with oligomeric procyanidins (OPCs) (natural phenolic compound) and lauric acid (LA) (lipid), was used for improving the mechanical, preservation, and barrier characteristics of gluten films [81]. The formed composite film exhibited a denser and compact structure, in addition to improving the tensile, film-forming, and oxygen-barrier properties, when compared to the control gluten film. Their results indicated that the edible composite film could potentially be applied as a packaging material for seasonings in the food industry as it maintained seasoning packaging capacity, to a certain degree, even after 75 days of storage.

### 4.9. As Emulsifier/Emulsion Stabilizer

FG can be used a substitute for common emulsifiers or stabilizers in emulsions. Some applications of FG as emulsifier/emulsion stabilizer are given in Table 3. FG was used as a stabilizer of emulsions manufactured with soybean oil and olive oil [82,83].

Owing to its emulsifying ability, FG was applied for decreasing the creaming of cloudy carrot juice [86]. In addition, the gum assisted the stabilization of the juice due to macromolecular steric repulsion.

Proteins are widely used to stabilize emulsions in the pharmaceutical and food industries. The heat denaturation of whey protein isolate (WPI) limits its application in foods. Doost et al. [87] showed that thermal-induced damage of WPI functionality can be prevented by conjugation (through dry heat treatment) to polysaccharides of the soluble fraction of flaxseed mucilage (SFM). On dry heat incubation, SFM formed covalent conjugates with WPI in a relatively short time. Among the studied ratios, a WPI-to-polysaccharide ratio of 1:1 was the most effective formulation in the formation of conjugates. The Maillard conjugation of WPI was found to increase the thermal stability of emulsions. Dong et al. [88] showed the superior emulsifying ability of FG–WPI conjugates prepared via Maillard reaction, especially those obtained by 72 h incubation. These conjugates exhibited significantly improved antioxidant activity.

Because of their water-thickening property, hydrocolloids can be used as stabilizers. Compared with protein-based emulsifiers, hydrocolloids have relatively low sensitivity to alterations in environmental conditions (i.e., ionic strength, pH, etc.). FG has the potential to substitute or integrate with protein emulsifiers, which are sensitive to pH changes, in emulsion-type products [89]. They showed a successful stabilization of oil-in-water emulsions (10% liquid lard) at pH 5.0 to 7.0 using FG at concentrations above 0.3% (*w*/*w*). Enhanced emulsifying and water-binding properties have been reported in certain food systems (i.e., meat emulsions, ice cream, and fish sauce) by the addition of linseed mucilage; meat emulsions formulated with linseed mucilage exhibited reduced firmness and reduced cooking loss [3].

Plant-based Pickering stabilizers (bioparticles) were developed for the purpose of stabilizing emulsions [90,91]. The bioparticles were fabricated from complexation (electrostatic interaction) of flaxseed protein (FP) and SFM. Using the selective negatively charged complex particles (369 nm size and neutral wettability), Pickering emulsions were fabricated. Compared with native FP, emulsions stabilized by the complex were stable with no droplet size variation during storage. They concluded that the adsorption of the FP–SFM complex nano-assemblies onto the interface introduced a protecting coating resisting coalescence and flocculation. These nano-assemblies displayed great potential as food-grade stabilizers for surfactant-free oil-in-water emulsions.

Flaxseed gum in combination with pea protein was used to coat the oil droplets (interfacial coating) in plant-based omega-3 oil emulsions [92]. The complex helped stabilize the emulsions (prevented emulsion aggregation and creaming). In addition, the layer inhibited lipid oxidation or reduced the release of off-flavor from the oil droplets. In a similar study, Xu et al. [93] showed an improvement in the physical stability of WPI-stabilized β-carotene emulsions with the addition of FG (as additional interfacial membrane) at a concentration of 0.1 wt.%. In addition, FG aided in the chemical stabilization of the emulsions and inhibited the degradation of β-carotene primarily by decelerating the mobility of the droplets.

### 4.10. Miscellaneous Applications

For sustained-release application, FM–chitosan polyelectrolyte complex (PEC) nanoparticles were developed via the ionic gelation method [94]. Nanometric particles with a size of 326 nm and a polydispersity index of 0.217 were prepared using FM and chitosan at concentrations of 0.011% (*w*/*v*) and 0.011% (*w*/*v*), respectively. These nanoparticles could be used for sustained or delayed release of food bioactive ingredients.

### 4.11. Limitations and Potential Solutions

In general, natural gums may exhibit some undesirable characteristics such as uncontrolled rates of hydration, thickening, microbial contamination, pH-dependent solubility, and viscosity loss with storage [95]. Some undesirable qualities of FG including the dull color of gum solution, low dissolution rate in cold water, and low storage stability limit its extensive use. Functional characteristics of FG may be enhanced via physical modification approaches (e.g., extrusion, micro-fluidization, and freeze–thaw cycling) and/or chemical modification approaches (e.g., oxidation, carboxymethylation, copolymerization, and thiolation) [96].

For a wider application of flaxseed gum in foods, the modification of flaxseed gum structure has been proposed [95]. The properties of flaxseed gum were modified and improved through the formation of carboxymethyl ethers. Following carboxymethylation, a more liquid-like property was noted with flaxseed gum, and this could be attributed to the reduced intermolecular association and suppressed entanglement of FG polysaccharide chains. Lower-viscosity carboxymethylated flaxseed gum could be favored in dietary fiber-fortified foods that enhance sensory characteristics without resulting in over-texturization.

## 5. Conclusions

Flaxseed mucilage has been shown to function as a nutraceutical. It also exhibits unique functional characteristics. In the present review, aspects related to characteristics, extraction approaches, and food applications of FM were discussed and summarized. Extraction parameters such as extraction temperature, pH of the extraction medium, and the addition of exogenous enzymes can significantly influence the chemical composition, yield, and functional characteristics of flaxseed gum. Additionally, novel technologies such as extrusion and ultrasonication can increase FM yield during extraction. FM has a wide range of food applications, predominantly as an encapsulating agent, emulsifier, and edible coating. Compared to synthetic materials, the merits of polysaccharide gums include biosafety, biodegradability, and sustainability. Physical and chemical modification of native FG could minimize its undesirable properties and enable its application for specific purposes. In general, the majority of plant seed-derived natural mucilages are nontoxic. However, toxicity screening is crucial if a new plant-derived mucilage is intended for use in food. Therefore, further studies are needed to evaluate the possible toxicity of FM toward humans.

## Figures and Tables

**Table 1:** Flaxseed gum (FG) as a polysaccharide of complex coacervates used for the encapsulation of unstable/volatile compounds.

Core Material	Wall/Shell Material	Findings	Reference
Flaxseed oil	Flaxseed protein isolate (FPI) and FG; the matrix was cross-linked with glutaraldehyde	1. Flaxseed oil was effectively microencapsulated by the crosslinked FPI–FG complex coacervates 2. Microencapsulation followed by spray-drying led to a doubling of oxidative stability of the oil	[61]
Flaxseed oil	Polyphenol-adducted FPI–FG complex coacervates; FPI was covalently adducted with flaxseed polyphenol (FPP) or hydroxytyrosol (HT)	1. The microcapsules produced with (FPI–HT)/FG complex coacervates exhibited the highest microencapsulation efficiency (95.4%) 2. The microcapsules encapsulated using (FPI–FPP)/FG complex coacervates possessed the highest oxidative stability	[62]
Vanillin/β-cyclodextrin inclusion	Rice bran protein–flaxseed gum (RBP–FG) coacervates	1. Vanillin/β-cyclodextrin-loaded microcapsules exhibited a maximum encapsulation efficiency of 85% at a protein-to-polysaccharide (Pr:Ps) ratio of 9:1, pH = 4.0, and core-to-wall ratio of 1:3 2. The thermostability and shelf life of vanillin were improved by microencapsulation	[63]

**Table 2:** Some reports of successful application of flaxseed mucilage as edible coating on food materials.

Coating Composition	Food Material	Findings	Reference
FM + alginate base containing probiotic bacteria *Lactobacillus casei* LC-01	Fresh-cut yacon	1. The edible coatings were found to be efficient carriers of the probiotic bacteria; viable cell counts of the microorganism were maintained at ~8 log CFU·g^−1^ of product throughout the storage 2. The viability of the bacteria was decreased, on average, by 2.96 log CFU·g^−1^ under simulated gastrointestinal conditions 3. The edible coatings aided in preserving the physicochemical characteristics of the vegetable, including the reduction of darkening and weight loss	[71]
Chitosan (CH), linseed mucilage (LM), and their combination (LMCH)	Fresh-cut cantaloupe	1. Effective for decreasing the softening and juice leakage 2. The combination (LMCH) reduced the microbicidal effect of CH 3. LM and CH ECs aided in the preservation of the overall sensory qualities, enhancing the product acceptance to 12–15 days 4. The combination (LMCH) assisted in preserving the characteristics of odor and color; however, it modified the taste and texture of fresh-cut cantaloupe, and its sensory acceptance was similar to the control (up to 9 days)	[72]
Layer-by-layer ECs based on CH + pullulan (PU), CH + LM, CH + nopal mucilage (NM), and CH + aloe mucilage (AM)	Fresh-cut pineapple	1. Increased the quality and extended the shelf life (by 6 days) 2. The use of the layer-by-layer ECs reduced (*p <* 0.05) the softening of pineapple tissue, weight loss, and delayed the decrease in color (L* and a*) and total soluble solid content	[73]
FG + lemongrass essential oil (LGEO)	Ready-to-eat pomegranate arils	1. These coatings aided in the maintenance of desired microbiological quality of the arils. 2. Upon increasing FG concentration to >0.6%, the coating solution became viscous, posing difficulties in applying the coating on the surface of any fresh-cut produce 3. However, the combinations FG (0.6%) + LGEO (500 ppm) and FG (0.6%) + LGEO (800 ppm) were found to be suitable for maintaining the sensory characteristics and other overall quality attributes	[74]
FM (0.75%, 1.0% and 1.25%) edible coatings	Cheddar cheese during ripening at 8 °C for 3 months	1. ECs insignificantly affected the growth of nonstarter lactic acid bacteria and total mesophilic aerobic bacteria 2. The coatings did not exert a significant influence on sensory attributes of cheddar cheese including color, texture, flavor, and cutting 3. However, the acidity, pH, TCA–SN/TN (trichloroacetic acid 12%–soluble nitrogen as percent of total nitrogen), and fat in dry matter of samples were significantly affected by FM treatment	[75]
A layer-by-layer electrostatic self-assembly technology was adopted to form ECs based on CH and FG	Mongolian cheese surface	1. The self-assembled coatings exhibited broad-spectrum bacteriostatic action 2. When chitosan was the innermost layer, it hindered the rapid growth of internal microorganisms (yeasts and molds), while it prevented the invasion of exotic microorganisms (*Staphylococcus aureus* and *Escherichia coli*) when it was the outermost layer 3. The technology not only enhanced the quality of Mongolian cheese but also prolonged its shelf life and delayed fat precipitation	[76]
Edible films based on FG + sodium alginate (SA) with varied concentrations of carvacrol	Chinese sea bass fillets during cold storage	1. The films containing carvacrol at concentrations of 1.0 or 2.0 mg/mL remarkably decreased the degree of microbial deterioration, total volatile basic nitrogen (TVB-N) content, and adenosine triphosphate (ATP) decomposition (*K* value), as well as maintained the quality (e.g., freshness) of sea bass fillets	[77]

**Table 3:** Some potential applications of flaxseed gum (FG) as emulsifier/emulsion stabilizer.

Flaxseed Gum Concentrations	Emulsion Type	Findings	Reference
0.1–0.5% *w*/*w*	Olive oil emulsions (oil-in-water)	1. FG enhanced the stability of emulsion with 10% olive oil 2. FG-stabilized emulsions exhibited a smaller droplet size, better rheological properties, and creaming stability	[83]
0.1–0.5% *w*/*w*	Soybean oil emulsions	1. With an increase in FG concentration, emulsion particle size decreased 2. FG exhibited gelling and thickening properties, and emulsions with 0.5% FG looked like a viscoelastic solid 3. Emulsions with a higher FG concentration exhibited superior structure and creaming stability	[82]
0.05–0.5% *w/v* + 1% *w/v* soybean protein isolate (SPI)	Soybean oil 10% *v/v* emulsion	1. At low concentrations (<0.1%) of FG, zeta potential and emulsion ability (turbidity) had similar decreasing trends 2. At 0.1% *w/v* FG concentration, emulsion particle size reached a minimum value 3. With gum concentrations >0.2%, emulsions exhibited larger particle size, higher zeta potential, and higher turbidity	[84]
0.3–0.6% *w*/*v*	Model oil-in-water salad dressings	1. FSB significantly contributed to the viscosity and stability of the emulsions 2. FSB concentration and pH were found to be critical factors determining the degree of emulsion stabilization 3. A highly stable emulsion was obtained at pH 4, 0.75% *w*/*w* flaxseed gum, and 2.5% *w*/*w* salt	[85]
0–0.33% *w*/*v*	WPI-stabilized oil-in-water emulsions at pH 3.5	1. Up to 0.1% FG concentration, the kinetic stability of the emulsions was noted 2. At FG concentrations >0.1%, bridging flocculation aided in the destabilization of the emulsion; at 0.2% FG, ζ-potential of the droplets decreased (from positive to a negative value) 2. Negatively charged polysaccharide fraction of FG interacted with the protein adsorbed at the interface 3. With the increase in FG concentration, apparent particle size increased	[31]

## Data Availability

Data is contained within the article.
